# Structure of Psb29/Thf1 and its association with the FtsH protease complex involved in photosystem II repair in cyanobacteria

**DOI:** 10.1098/rstb.2016.0394

**Published:** 2017-08-14

**Authors:** Martina Bec̆ková, Jianfeng Yu, Vendula Krynická, Amanda Kozlo, Shengxi Shao, Peter Koník, Josef Komenda, James W. Murray, Peter J. Nixon

**Affiliations:** 1 Institute of Microbiology, Center Algatech, Opatovický mlýn, 37981 Třeboň, Czech Republic; 2 Faculty of Science, University of South Bohemia, Branišovská 1760, 370 05 České Budějovice, Czech Republic; 3 Sir Ernst Chain Building-Wolfson Laboratories, Department of Life Sciences, Imperial College London, South Kensington Campus, London SW7 2AZ, UK

**Keywords:** photoinhibition, thylakoid formation 1 gene, D1 subunit, *Synechocystis*, thylakoid membrane, hypersensitive response

## Abstract

One strategy for enhancing photosynthesis in crop plants is to improve their ability to repair photosystem II (PSII) in response to irreversible damage by light. Despite the pivotal role of thylakoid-embedded FtsH protease complexes in the selective degradation of PSII subunits during repair, little is known about the factors involved in regulating FtsH expression. Here we show using the cyanobacterium *Synechocystis* sp. PCC 6803 that the Psb29 subunit, originally identified as a minor component of His-tagged PSII preparations, physically interacts with FtsH complexes *in vivo* and is required for normal accumulation of the FtsH2/FtsH3 hetero-oligomeric complex involved in PSII repair. We show using X-ray crystallography that Psb29 from *Thermosynechococcus elongatus* has a unique fold consisting of a helical bundle and an extended C-terminal helix and contains a highly conserved region that might be involved in binding to FtsH. A similar interaction is likely to occur in Arabidopsis chloroplasts between the Psb29 homologue, termed THF1, and the FTSH2/FTSH5 complex. The direct involvement of Psb29/THF1 in FtsH accumulation helps explain why THF1 is a target during the hypersensitive response in plants induced by pathogen infection. Downregulating FtsH function and the PSII repair cycle via THF1 would contribute to the production of reactive oxygen species, the loss of chloroplast function and cell death.

This article is part of the themed issue ‘Enhancing photosynthesis in crop plants: targets for improvement’.

## 1. Introduction

Plants exposed to excessive light suffer from impaired photosynthetic activity termed chronic photoinhibition [1,2]. One of the main targets of damage is the oxygen-evolving photosystem II (PSII) complex embedded in the thylakoid membrane system, which uses light energy to extract electrons from water to feed into the photosynthetic electron transport chain to produce the ATP and NADPH required for CO_2_ fixation [3]. Irreversible inactivation of PSII occurs at all light intensities [4,5], but activity can be restored through the operation of a repair cycle that replaces damaged protein subunits, mainly the D1 reaction centre subunit, with a newly synthesized copy [1,6]. Only when repair cannot match damage is there a net loss of PSII activity. Consequently, improving the efficiency of the repair cycle, which itself is susceptible to oxidative damage [7], is a potential route to enhance photosynthesis in crop plants exposed to light stress.

Repair of PSII occurs in all organisms that carry out oxygenic photosynthesis [8,9]. Although there are some differences in the structures of PSII in cyanobacteria and chloroplasts [10], many of the accessory factors and proteases involved in PSII assembly and repair are conserved [11,12], making cyanobacteria extremely useful models to study the molecular details of PSII biogenesis [13].

The main pathway for degrading damaged D1 during repair involves proteolysis by specific members of the FtsH family of ATP-dependent metalloproteases in both cyanobacteria [14,15] and chloroplasts [16–18]. In the case of the cyanobacterium *Synechocystis* sp. PCC 6803 (hereafter *Synechocystis* 6803), electron microscopy has revealed the isolated FtsH complex to be hexameric and composed of alternating FtsH2 and FtsH3 subunits [19], which, based on phylogenetic analyses, have been classified as type B and type A FtsH isoforms, respectively [20,21]. Although structural confirmation is currently lacking, similar hexameric hetero-complexes consisting of type A and type B subunits are likely to be involved in PSII repair in chloroplasts [18,21], with the dominant complex in Arabidopsis composed of FTSH2 (a type B subunit orthologous to FtsH2) and FTSH5 (a type A subunit orthologous to FtsH3) [21]. The Arabidopsis FTSH2 and FTSH5 subunits are also called VAR2 and VAR1, respectively, due to the yellow variegated phenotype of the *var2* and *var1* null mutants [21]. As the chloroplast FtsH proteases are nuclear-encoded in Arabidopsis, the gene products are written in uppercase and the mutants in lower case and in italics.

How expression of FtsH complexes is regulated in response to light stress is unclear. Recent studies of the variegated *thf1* (*thylakoid formation 1*) mutant of Arabidopsis [22] have indicated that the THF1 protein is required for normal accumulation of FTSH2/VAR2 and FTSH5/VAR1 and that this effect is post-transcriptional [23,24]. The THF1 homologue in cyanobacteria, designated Psb29 or Thf1, was originally identified as a sub-stoichiometric component of isolated His-tagged PSII preparations of *Synechocystis* 6803 [25] and a role in the maintenance of PSII was suggested on the basis of the enhanced sensitivity of PSII activity to light stress in a *Synechocystis* 6803 *psb29* null mutant, but specific effects on FtsH were not examined [26]. A reduction in the level of FtsH was recently reported in a *psb29* null mutant of the cyanobacterium *Synechococcus* sp. PCC 7942, but changes to the expression of individual FtsH subunits were not investigated [27]. In addition it has been proposed that Psb29/Thf1 interacts with photosystem I [27].

Here we show that Psb29 in *Synechocystis* 6803, like THF1 in Arabidopsis, is important for normal accumulation of the FtsH heterocomplex involved in PSII repair. Furthermore, affinity purification data suggest that Psb29 physically interacts with FtsH complexes *in vivo*. To gain further insights into Psb29, we have determined the crystal structure of Psb29 encoded by *Thermosynechococcus elongatus*, a thermophilic cyanobacterium widely used to study structural aspects of PSII assembly and repair [28,29]. Psb29 contains a highly conserved surface on one face of the molecule that might be important for specific protein/protein interactions such as with FtsH. A striking feature of Psb29 is the presence of a long alpha helix at the C-terminus extending from the globular protein domain.

## 2. Material and methods

### (a) Cyanobacterial strains and growth conditions

All mutants were constructed in the glucose-tolerant WT-P strain of *Synechocystis* sp. PCC 6803 [30] and grown using BG11 medium as described in [31]. For mixotrophic cultivation, glucose was normally added to 5 mM. For protein and RNA analyses, 50–100 ml liquid cultures of *Synechocystis* 6803 were grown on an orbital shaker in BG11 medium in 250 ml conical flasks at 29°C under moderate light conditions (40 µmol photons m^−2^ s^−1^). For purification of protein complexes, the FtsH2-FLAG strain was grown as described above in 500 ml of medium using 2 l conical flasks. For purification of Psb29-FLAG protein complexes, 4 l of Psb29-FLAG strain were grown in a 10 l flask in BG11 medium supplemented with 1 mM glucose, agitated with magnetic stirrer and bubbled with air. In both cases, surface irradiance was increased to 100 µmol photons m^−2^ s^−1^ of light to compensate for the longer path length of the flasks. For spot growth tests, 2.5 µl of mixotrophic culture and 10^2^, 10^3^ and 10^4^ serial dilutions were spotted onto BG11 agar plates and grown for 7 days.

### (b) Construction of cyanobacterial mutants

The transformation vector for disruption of *psb29* gene in *Synechocystis* 6803 (Cyanobase designation *sll1414*) was constructed in two steps. First, the flanking sequencing of *sll1414*, 445 bp upstream and 555 bp downstream, was PCR amplified with primer set sll1414-1F (AGTTTCTCGTTCTGCCGCCTCAGCTCTT) and sll1414-2R (AATGGGGCCTCATAGTGGGGCATGGATTGAAGATATCAGGGCCGATTACAAAGGGGGGGATAGT), and sll1414-3F (ACTATCCCCCCCTTTGTAATCGGCCCTGATATCTTCAATCCATGCCCCACTATGAGGCCCCATT) and sll1414-4R (ATTAACTCCCCATCCACTTCCACTTCGATGAT). The resulting PCR products were then mixed as DNA template for overlap extension PCR with primer set sll1414-1F and sll1414-4R. The fused PCR fragment containing an EcoRV restriction site instead of *sll1414* ORF was then cloned into pGEM-T Easy vector. In the second step, a DNA cassette that confers chloramphenicol resistance was inserted into the EcoRV site. Two transformation vectors were selected due to the nature of blunt-end ligation: pSll1414camA has the chloramphenicol marker integrated in the same direction as *sll1414*, whereas, pSll1414camB has the marker in the opposite direction. Both plasmids were used to transform the glucose-tolerant WT-P strain of *Synechocystis* 6803, yielding strains ΔPsb29camA and ΔPsb29camB.

The transformation vectors for expressing C-terminal 3xFLAG-tagged derivatives of Psb29 and FtsH2 at the *psbA2* locus were generated by cloning PCR fragments into the NdeI and NheI sites of pPD-CFLAG [32]. The coding sequence of *psb29* (*sll1414)* was amplified with primer pair CF-Psb29-F (TTTTTTCATATGACTAAAATTCGCACTGTTTCTGACGCCAA) and CF-Psb29-R (TTTTTTGCTAGCGCTTTCGGAACTCTCCGCTGTGGTT) and the coding sequence of *ftsH2* (*slr0228*) was amplified with primer set CF-FtsH2-F (TTTTTTCATATGAAATTTTCCTGG.AGAACTGCCCTACTT) and CF-FtsH2-R (TTTTTTGCTAGCTAGTTGGGGAATTAACTGTTCCTTGACGGGA). The *Synechocystis* 6803 mutant ΔPsb29camA was used as background strain to generate Psb29-FLAG/ΔPsb29 and insertion mutant *slr0228::cm^R^* [15] was transformed to generate FtsH2-FLAG/ΔFtsH2.

### (c) Preparation of membranes, FLAG-tag immunoaffinity purification and protein analysis

Preparation of membranes by breaking cells using a Mini-Beadbeater-16 (BioSpec) and anti-FLAG pull downs were performed as described in [33]. The chlorophyll concentration of cells and various preparations was measured by extracting into methanol and measuring the absorbances at 666 and 720 nm [34]. Analysis of protein complexes was performed using two dimensional clear-native/SDS polyacrylamide gel electrophoresis (2D-CN/SDS PAGE) on a 4 to 14% native and 12 to 20% SDS gel containing 7 M urea, respectively [33]. The gels were stained either with Coomassie Blue and the visualized bands subjected to mass spectrometric (MS) analysis or with the fluorescence dye SYPRO Orange, then blotted onto PVDF membrane for immunodetection. Proteins were detected using antibodies specific for FtsH1, FtsH2, FtsH3 and global FtsH (FtsHg) [19], Phb1 and Phb3 [35] and Psb29 using an antiserum raised against a peptide corresponding to residues 155–172 of *Synechocystis* Psb29 conjugated to keyhole limpet haemocyanin (Clonestar, Brno, Czech Republic).

### (d) Mass spectrometric identification of proteins

The MS analyses of protein bands excised from gels were done on a NanoAcquity UPLC (Waters) on-line coupled to an ESI Q-ToF Premier mass spectrometer (Waters), as described in [36].

### (e) Determination of *ftsH2* and *ftsH3* transcript levels

Determination of the *ftsH2* and *ftsH3* transcript levels by quantitative PCR was performed as described in [31] using specific primers for *ftsH2* and *ftsH3* and Transcriptor Reverse Transcriptase (Roche). The *rnpB* gene encoding the B subunit of ribonuclease P was used as a reference and the analysis was performed in triplicate using three independent cultures.

### (f) Expression of Psb29 and structure solution

The coding sequence of Psb29 from *T. elongatus* (Cyanobase designation: Tlr1134) was cloned into the BamHI and XhoI sites of the modified pRSETA expression vector [28] following amplification of *psb29* using primer set Tlr1134-F (GGATCCGTGCAAAATCCTCGAACTGTCTCTGATACCAAACG) and Tlr1134-R (CTCGAGTCAAGCGGGTGCATCGGAGCTGGCAT). The resulting vector pRSETAPsb29 encodes a recombinant protein consisting of a 6xHis tag at the N-terminus followed by a thrombin cleavage site then Psb29. The *E. coli* strain KRX was used for recombinant Psb29 expression. Psb29 expression in transformed cells was induced at an OD_730_ of 0.8 with 1 g l^−1^ rhamnose and cells were then grown at 18°C overnight. Cells were lysed by sonication in lysis buffer (50 mM Tris-HCl pH 7.9, 500 mM NaCl, 1 mM MgCl_2_). In some preparations, the lysis buffer was supplemented with a Complete Protease Inhibitor Cocktail Tablet – EDTA (Roche, UK). The supernatant was mixed with a Ni-IDA resin (Generon, UK). Non-specifically bound proteins were removed by washing three times with wash buffer (20 mM Tris-HCl pH 7.9, 500 mM NaCl, 60 mM imidazole) and Psb29 was eluted with elution buffer (20 mM Tris-HCl pH 7.9, 500 mM NaCl, 1M imidazole). The protein was concentrated to around 10 mg ml^−1^ in 20 mM Tris-HCl pH 7.9, 500 mM NaCl and used for crystallization trials. Concentrated samples were placed in sitting drop vapour diffusion crystallization screens using a Mosquito^®^ robot (TTP LabTech, UK).

For preparations in the presence of protease inhibitor, the only crystals obtained were of needle morphology in P6_3_22, which diffracted very weakly. If protease inhibitor was omitted, crystals were readily obtained in three crystal forms. Two of these were in P2_1_ (designated A-P2_1_ and B-P2_1_) and the third in I222. The structure was solved by single-wavelength anomalous dispersion (SAD) with the A-P2_1_ crystal form, soaked overnight with 1 mM dipotassium tetraiodomercurate (Jena Bioscience). The P6_3_22 form was soaked overnight in 1 mM 4-(Chloromercuri)benzensulfonic acid sodium salt (Jena Bioscience), but this was not used for phase determination. Crystals were cryoprotected in the mother liquor with 30% glycerol added, and flash-cooled in a loop into liquid nitrogen. Diffraction data were collected at Diamond Light Source and processed using xia2 [37] with XDS [38]. See table 1 for data collection and refinement information. Heavy atom sites for A-P2_1_ were found and the structure phased using the autoSHARP [39] pipeline. The initial model was built with Buccaneer [40] and refined with REFMAC [41]. The B-P2_1,_I222 and P6_3_22 crystal forms were solved by molecular replacement with Phaser [42] using the A-P2_1_ structure as a model. These structures were refined with REFMAC or phenix.refine [43]. Structures were validated using MolProbity [44].

**Table 1.** Data collection and refinement statistics for the Psb29 structures. Values in brackets refer to the high resolution shell. DLS, Diamond Light Source.

crystal form	P6322	A-P21	B-P21	I222
PDB	5MLF	5MJO	5MJR	5MJW
structure	Psb29 full-length	Psb29 truncated	Psb29 truncated	Psb29 truncated
crystallization condition	16% w/v PEG 6 K, 80 mM sodium citrate pH 5	0.1 M Bicine pH 9.0, 20% w/v PEG 6 K	0.1 M sodium citrate pH 5, 20% w/v PEG 6 K	0.2 M sodium malonate pH 7, 20% w/v PEG 3350
beamline	DLS I03	DLS I04-1	DLS I04-1	DLS I03
wavelength (Å)	0.9537	0.91730	0.91730	0.97630
space group	P6_3_22	P2_1_	P2_1_	I222
unit cell a,b,c (Å) α, β, γ (°)	138.91,138.91,205.91, 90,90,120	31.240, 56.730, 47.730, 90, 104.720,90	39.640, 56.040, 44.530 90, 105.760, 90	62.850, 86.610, 116.020, 90, 90, 90
resolution	56–3.64 (3.73–3.64)	46–1.55 (1.59–1.55)	42.9–1.38 (1.42–1.38)	55.3–2.47 (2.53–2.47)
total no. reflections	278837 (21276)	392216 (28397)	259805 (18416)	73967 (5401)
no. unique reflections	20447 (1466)	23219 (1702)	38130 (2813)	11662 (838)
completeness (%)	99.92 (100.0)	99.0 (99.2)	98.7 (98.3)	99.6 (98.8)
multiplicity	13.6 (14.5)	16.9 (16.7)	6.8 (6.5)	6.3
<I/sigmaI>	7.4 (4.5)	18.4 (3.4)	16.0 (2.8)	16.5 (3.4)
Rmerge	0.394 (0.764)	0.114 (1.098)	0.071 (0.796)	0.062 (0.827)
Wilson B (Å^2^)	17.2	15.9	13.1	73.2
refinement				
program	phenix.refine	refmac	refmac	refmac
% test set	5.13	5.1	5.0	4.8
*R*_cryst_	0.3110	0.13321	0.11356	0.21760
*R*_free_	0.3575	0.19243	0.15544	0.24978
RMS				
bonds (Å)	0.002	0.024	0.026	0.008
angles (°)	0.471	2.070	2.181	1.191
Ramachandran plot (molprobity)				
most favoured (%)	96.52	98.91	98.37	96.37
outliers (%)	0	0	0	0.52

### (g) Bioinformatics

211 Psb29 sequences were retrieved by blasting Psb29 from *Synechocystis* 6803 (*sll1414* gene product) against UniProt KnowledgeBase Reference proteomes (http://www.uniprot.org). The cut-off threshold was empirically set to 1×10^−4^ after manually examining the resulting hits. 103 records were from cyanobacteria, 84 from plant, 11 from green algae, 12 from red algae and one from a virus that infects the green alga *Chlorella* sp. strain NC64A. 211 sequences were then aligned using MAFFT version 7 programme with the ‘G-INS-I’ setting applied [45]. Gaps within the alignment were trimmed by trimAl using the ‘gappyout’ method [46] and then the alignment was subjected to maximum-likelihood based phylogenetic inference, PhyML. ETE3 toolkit [47] was used to automate the above process; the PhyML setting was ‘+G+I+F, 4 classes and aLRT branch supports, default models JTT/GTR’ [48]. The final unrooted tree was organized and beautified with iTOL [49]. Subsets of 103 cyanobacterial and 84 plant Psb29 sequences were clustered according to their phylogeny. The trimmed alignments used in the conservation analysis were subjected to identity and similarity calculations using MatGAT [50]. The evolutionary conservation was analysed using ConSurf 2016 server [51]. The above MAFFT alignment was trimmed of columns containing gaps of over 90%; columns corresponding to the chloroplast transit peptide domain of *Arabidopsis thaliana* THF1, predicted by ChloroP 1.1 Server [52], were also removed.

## 3. Results

### (a) Psb29 is required for normal expression of FtsH2 and FtsH3 in *Synechocystis* 6803

**Figure 1.**
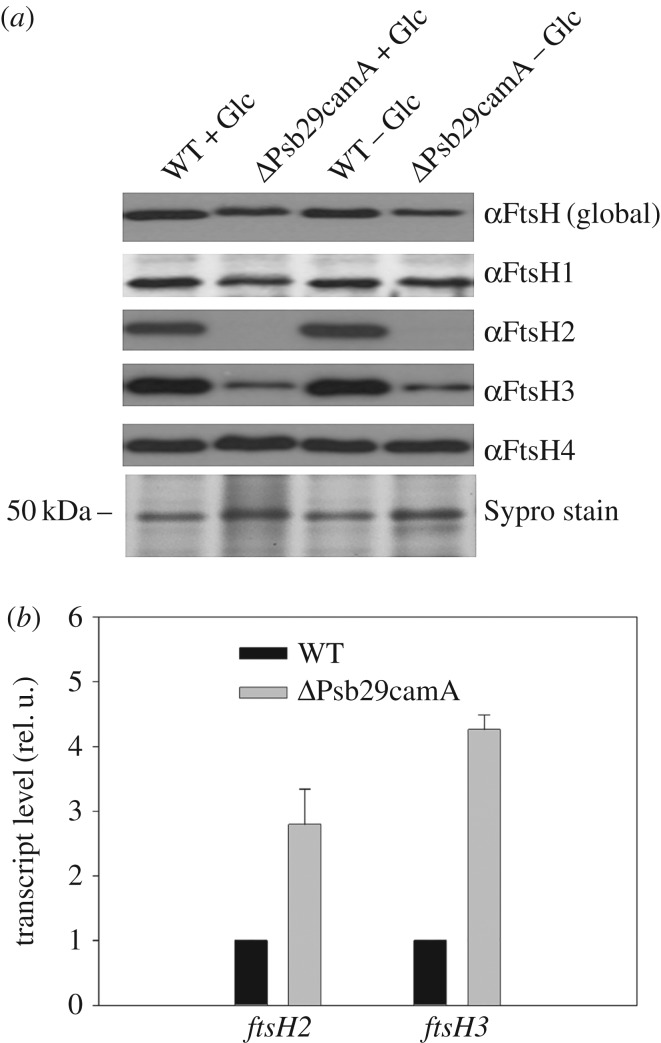
(*a*) Immunochemical analysis of FtsH subunits in WT and ΔPsb29camA grown either in the presence (+Glc) or absence (−Glc) of glucose until an OD_730_ of 0.6–0.8. Protein loading assessed by protein staining (Sypro stain). (*b*) Relative transcript levels of *ftsH2* and *ftsH3* in WT and ΔPsb29camA determined by RT-PCR.

To test whether Psb29 plays a role in the expression of FtsH in *Synechocystis* 6803, we performed an immunoblotting analysis of membranes isolated from a *psb29* null mutant, ΔPsb29camA, in which the *psb29* gene was replaced by a chloramphenicol-resistance cassette (electronic supplementary material, figure S1*a*,*b*). Cultures grown to late-exponential phase under either photoautotrophic or mixotrophic conditions were analysed. Antibodies specific for each of the four FtsH proteins encoded by *Synechocystis* 6803 revealed that levels of FtsH2 and FtsH3 were decreased substantially in the mutant compared to the WT control, consistent with a specific effect on the accumulation of the FtsH2/FtsH3 hetero-complex, whereas there was less of an impact on FtsH1 and FtsH4 (figure 1*a*). Similar results were also obtained with a *psb29* null mutant, ΔPsb29camB, containing the chloramphenicol-resistance cassette inserted in the opposite orientation (electronic supplementary material, figure S1*a–c*). Reverse-transcription PCR confirmed that *ftsh2* and *ftsH3* were still transcribed in ΔPsb29camA so the effect of Psb29 on the expression of FtsH2 and FtsH3 occurred after transcription (figure 1*b*). The 2–5-fold increase in *ftsH2* and *ftsH3* transcripts in ΔPsb29camA might reflect a compensatory mechanism to increase expression. Importantly, immunoblotting experiments showed that FtsH2 and FtsH3 expression was reduced but not blocked totally in the absence of Psb29 (electronic supplementary material, figure S1*c*).

### (b) Psb29 interacts with FtsH complexes

**Figure 2.**
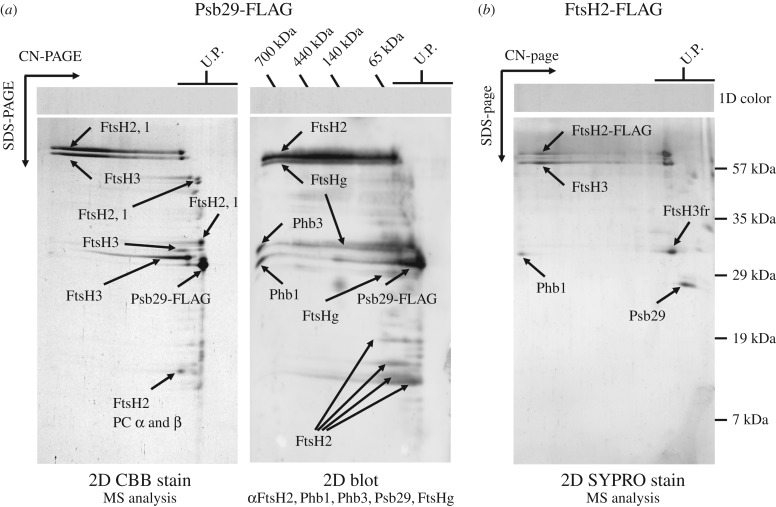
(*a*) Isolation of FLAG-tagged Psb29 and identification of co-purifying proteins by 2D gel electrophoresis followed by Coomassie Brilliant Blue (CBB) staining and mass spectrometry (left panel) or by sequential immunochemical detection with antibodies in the order shown starting with FtsH2 (right panel). The global FtsH antibody recognizes all FtsH isoforms (FtsHg) whereas the other FtsH antibodies are specific for each subunit. (*b*) Isolation of FLAG-tagged FtsH2 and detection of proteins by mass spectrometry after staining gel with Sypro orange (SYPRO stain).

To test whether Psb29 interacts with FtsH we generated two strains of *Synechocystis* 6803 expressing either Psb29 or FtsH2 tagged at the C-terminus by addition of a 3XFLAG tag. Expression of the tagged proteins under the control of the *psbA2* promoter in the relevant *ftsH2* or *psb29* null mutant restored photoautotrophic growth at high irradiances, indicating that the tagged proteins were still functional (electronic supplementary material, figure S2). Immunoaffinity purification of Psb29-FLAG from detergent-solubilised membranes using anti-FLAG antibodies, followed by 2D gel electrophoresis (clear-native in the first dimension and denaturing in the second) and detection of proteins by protein staining, immunoblotting and mass spectrometry revealed the presence of large complexes containing FtsH2, FtsH3 and FtsH1 (figure 2*a*), which we assign to FtsH2/FtsH3 and FtsH1/FtsH3 heterocomplexes based on previous studies [19]. Also detected were minor amounts of fragments derived from FtsH1, FtsH2 and FtsH3 that migrated as unassembled proteins, and two members of the Band 7 superfamily: prohibitin (Phb1) previously detected in FtsH2/FtsH3 preparations [19] and Phb3 [35]. Psb29-FLAG did not co-migrate with FtsH in the native gel, suggesting detachment during electrophoresis. The reciprocal immunoaffinity purification using the FtsH2-FLAG strain confirmed the co-purification of Psb29 with FtsH2 and FtsH3 (figure 2*b*). Overall these data support the direct interaction of Psb29 with FtsH2/FtsH3 complexes.

### (c) Crystal structure of Psb29 from *T. elongatus*

**Figure 3.**
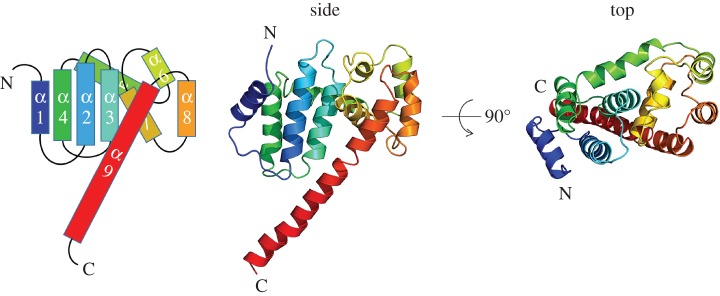
Structure of Psb29 (PDB: 5MLF) encoded by *T. elongatus* showing side and top views and cartoon representation of the 9 alpha helices.

To gain structural information on Psb29, we over-expressed Psb29 encoded by the cyanobacterium *T. elongatus* as an N-terminal His-tagged protein in *E. coli* and isolated the protein by Ni-affinity chromatography. Four crystal forms were obtained by hanging drop vapour diffusion; X-ray diffraction data were collected at resolutions from 3.6 Å to 1.4 Å and the structure of Psb29 determined by heavy atom SAD (table 1). The most complete structure consisting of residues 4 to 206 of the predicted 222 residues of Psb29 was obtained from P6_3_22 needle-shaped crystals containing seven copies of Psb29 in the asymmetric unit, which form a continuous cylindrical shell of protein in the crystal, with the C-terminus of the protein forming a helix extending from the compact protein fold into the middle of the cylindrical protein shell (electronic supplementary material, figure S3*a*). Each Psb29 subunit consists of 9 alpha helices (figure 3). A search using PDBeFOLD [53] found no known structures with greater than 70% similarity, indicating that the specific fold is novel.

Psb29 in the other crystal forms was proteolytically cleaved at the C-terminus. In the B-P2_1_ crystal form, the new carboxy terminus at residue Ala189 is clearly visible in the electron density (electronic supplementary material, figure S3*b*). It is likely that proteolytic cleavage of the C-terminal helix allows more compact higher resolution crystal lattices to form, as there is insufficient space in these lattices to accommodate the C-terminal helix observed in the P6_3_22 crystal form. The I222 crystal form shows a domain-swapping of the N-terminal helix from the N-terminus to residue Ile22, creating a domain-swapped dimer (electronic supplementary material, figure S3*c*). Given that the domain-swap is not observed in the other crystal forms, this is probably a crystallization artefact.

### (d) Comparison of Psb29/THF1 sequences

Bioinformatic analyses revealed that Psb29 and its eukaryotic homologue THF1 are found solely in oxygenic photosynthetic organisms (electronic supplementary material, figure S4). One exception is a virus infecting the green alga *Chlorella* sp. strain NC64A that possesses a Psb29-encoding gene closely related to green algal Psb29 sequences (electronic supplementary material, figure S4). In the proteome database interrogated on 11th November 2016, 103 out of 106 cyanobacteria were found to encode Psb29 homologues. The genome sequences of the three remaining cyanobacteria, *Limnoraphis robusta* CS-951, *Leptolyngbya valderiana* BDU 20041, and *Cyanobium* sp. PCC 7001 (*Synechococcus* sp. PCC 7001) are still incomplete and so still yet might encode Psb29.

**Figure 4.**
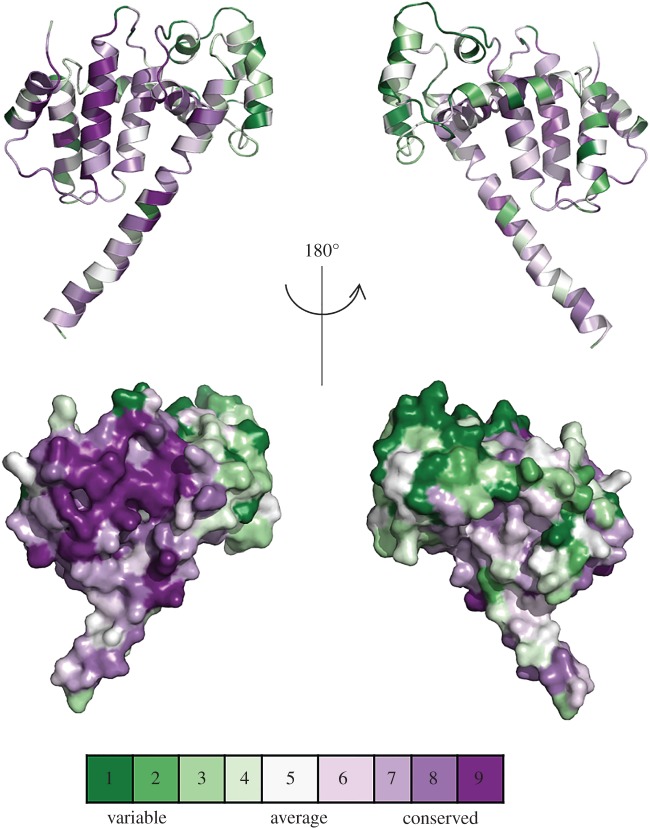
Highly conserved residues in *T. elongatus* Psb29. A ConSurf analysis was performed based on the alignment of 211 Psb29/THF1 sequences from oxygenic phototrophs. The front and back views highlight the conserved and variable regions of Psb29 using the following colouring scheme: purple, 9 = maximal conservation; white, 5 = average conservation; green, 1 = maximal variability.

**Figure 5.**
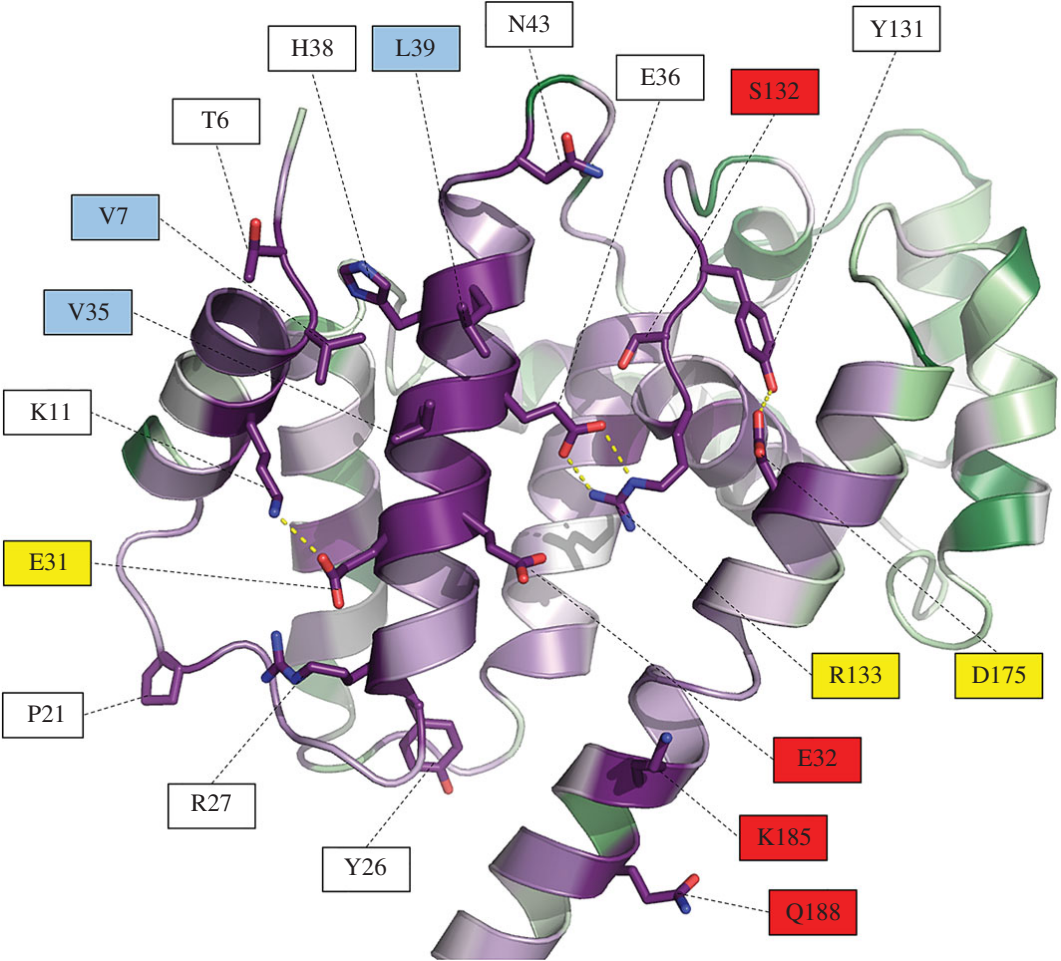
Close-up view of the conserved residues of Psb29/THF1 identified by ConSurf analysis. The most conserved residues that are not buried within the Psb29 structure are shown in stick form, with red indicating oxygen atoms and blue nitrogen atoms. Intra-protein side-chain polar contacts are shown as yellow dashed lines. Some residues are colour-coded to indicate possible type of interaction. Red labels indicate potential hydrogen bonding/charged residues that might stabilize protein/protein interactions; yellow labels indicate residues possibly involved in both stabilizing the structure and interacting with proteins; light blue labels indicate potential hydrophobic contact sites.

Overall Psb29 from *T. elongatus* shows a mean sequence similarity of 59.2% with the 102 cyanobacterial Psb29 sequences examined and 53.7% with the 84 plant THF1 sequences. Six residues are totally conserved in cyanobacterial and plant Psb29/THF1 sequences (electronic supplementary material, figure S5): based on the structure described here, F14, V35, L39, G55 and G138 (*T. elongatus* numbering) appear important for the packing of alpha helices and R133 at the beginning of helix 7 is within H-bonding distance of E36 in the middle of helix 2 (electronic supplementary material, figure S4). These sequence identities would suggest a high degree of conservation of tertiary structure between Psb29 and THF1 in this region of the molecule. A ConSurf analysis in which all Psb29/THF1 sequences were fitted into the *T. elongatus* structure revealed high sequence conservation on one face of the molecule, which would indicate an important role for this region in protein function (figure 4). There are several conserved residues in this region that might play a role in binding interacting partners such as FtsH (figure 5).

The alignment of Psb29/THF1 sequences revealed a variety of small insertions and deletions. In the case of plant THF1, these insertion/deletion events correspond to *T. elongatus* residues 121–122 and 151–154, which lie in loop regions connecting alpha helices 6–7 and 7–8, respectively (electronic supplementary material, figure S5), in the more divergent region of Psb29. The C-terminal end of the protein is also poorly conserved (electronic supplementary material, figures S5 and S6).

## 4. Discussion

Previous work in Arabidopsis has shown that the absence of THF1 leads to a 40–80% decrease in the amount of the type A and type B FTSH subunits involved in PSII repair as judged by immunoblotting [23]. We show here that loss of Psb29 has a similar effect in cyanobacteria, as levels of the FtsH2 and FtsH3 subunits that form the FtsH heterocomplex involved in PSII repair in *Synechocystis* 6803 are likewise reduced in *psb29* null mutants (figure 1; electronic supplementary material, figure S1*c*). These data suggest a conserved role for Psb29/THF1 in fine-tuning the expression of thylakoid FtsH heterocomplexes.

Importantly, we have provided evidence that Psb29 interacts directly with FtsH2/FtsH3 complexes (figure 2). Thus we suggest that Psb29/THF1 plays a direct role in the accumulation of FtsH heterocomplexes. Based on the co-purification of FtsH1 with Psb29-FLAG (figure 2*a*), it is possible that Psb29 is also involved in the accumulation of FtsH1/FtsH3 heterocomplexes [19]. However, levels of FtsH1 were much less affected than FtsH2 and FtsH3 in the *psb29* null mutant under the conditions examined (figure 1).

Recent work, based on the analysis of cross-linked membrane protein complexes by sucrose density gradient centrifugation, has concluded that Psb29 in the cyanobacterium *Synechococcus* sp. PCC 7942 binds to PSI complexes [27]. However, pull-down experiments were not done to confirm cross-linking between Psb29 and PSI. In light of our data, we suggest that further work is needed to exclude the possibility that Psb29 is actually cross-linked to FtsH complexes, which then co-sediment with PSI. Reduced expression of PSI was also reported in a *psb29* null mutant [27] but this might be related to effects on expression of FtsH2 rather than a direct effect of Psb29 [54].

We have also presented the first structural information on Psb29. The first 3 and last 16 residues could not be identified in the most complete crystal structure, possibly because of structural flexibility or because of some proteolytic degradation. The fitting of cyanobacterial and plant Psb29/THF1 proteins into the *T. elongatus* crystal structure using ConSurf has allowed us to identify a highly conserved surface on Psb29 that might be involved in protein/protein interactions, such as with FtsH (figures 4 and 5). Recent work has indicated that residues 223–295 of THF1 of *Nicotiana benthamiana*, encompassing part of helix 8, all of helix 9 and most of the C-terminal tail, is a target for a sub-group of nucleotide-binding leucine-rich-repeat (NB-LRR) proteins involved in plant immunity [55]. Thus some of the observed sequence variation between Psb29 and THF1 might reflect changes in THF1 function since the divergence of plants and cyanobacteria.

PSII repair is one of several photoprotective mechanisms used by plants [2]. Despite its physiological importance, little work has been directed at enhancing PSII repair in crop plants, either in terms of robustness or speed of response. In the case of plants, damaged PSII complexes must migrate from the appressed membranes in the grana to the margins to be repaired [56]. This means that prompt degradation of damaged D1 might become a bottleneck in the repair process and that enhancing the expression of FTSH proteases, or DEG proteases that act as a second-line of defence [17], might delay or prevent chronic photoinhibition. Our work now identifies Psb29/THF1 as an additional target for manipulation.

Work in cyanobacteria has highlighted D1 synthesis as a weak link in PSII repair due to reactive oxygen species (ROS)-mediated oxidation of elongation factor EF-G required for protein translation [57]. Attempts to improve protein synthesis by mutating the two Cys residues of EF-G sensitive to oxidative damage has had limited success [58]. Instead a more promising approach is the over-expression of enzymes to detoxify ROS [59]. Prompt replacement of D1 during repair might also be helped by increasing the pool of unassembled D1 in the membrane that could be tapped into to replace damaged D1. One approach might be to over-express the higher plant homologues of Ycf48 and the Ycf39/Hlip complex, which have been shown to stabilize unassembled D1 in cyanobacteria [60,61].

Although upregulating FtsH activity and the PSII repair cycle would seem beneficial for plant growth, there appear to be situations where plants deliberately downregulate chloroplast FtsH activity, which is known to lead to the enhanced production of ROS even under non-photoinhibitory conditions [62]. The source of ROS is not clear but they could be produced by defective PSII complexes that have not been promptly repaired. One dramatic example is the hypersensitive response (HR), which is induced to kill plant cells infected by pathogens so as to limit the zone of infection [63]. Although chloroplast FtsH had previously been implicated in HR [64], the mechanism has been unclear. Recent evidence has suggested a role for THF1 in the signal transduction pathway [55,65]. Our data would suggest that loss of THF1 in the chloroplast plays a direct role in the decrease of FtsH activity, either by destabilizing FtsH complexes, as observed in the Arabidopsis *thf1* null mutant [23] and/or by impairing assembly. Evidence from both cyanobacteria [66] and *Chlamydomonas reinhardtii* [67] suggests that upregulating synthesis of FtsH is important for acclimation to higher light intensities as well as possibly replacing damaged FtsH.

## Supplementary Material

Supplementary figures

## Data accessibility

This article has no additional data.
