# Supplementary material for: Structure of Psb29/Thf1 and its association with the FtsH protease complex involved in photosystem II repair in cyanobacteria
Source: Philos Trans R Soc Lond B Biol Sci. 2017 Aug 14;372(1730):20160394. doi: 10.1098/rstb.2016.0394 (PMC5566888; doi:10.1098/rstb.2016.0394)
Supplement: Supplementary figures [file rstb20160394supp1.pdf]

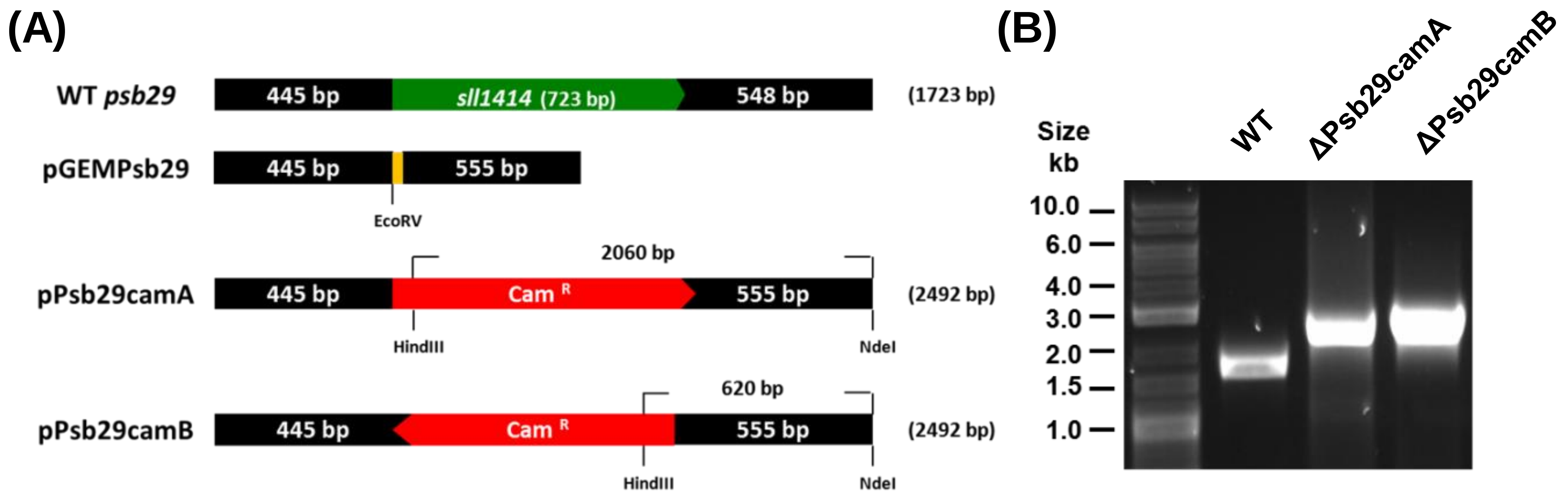

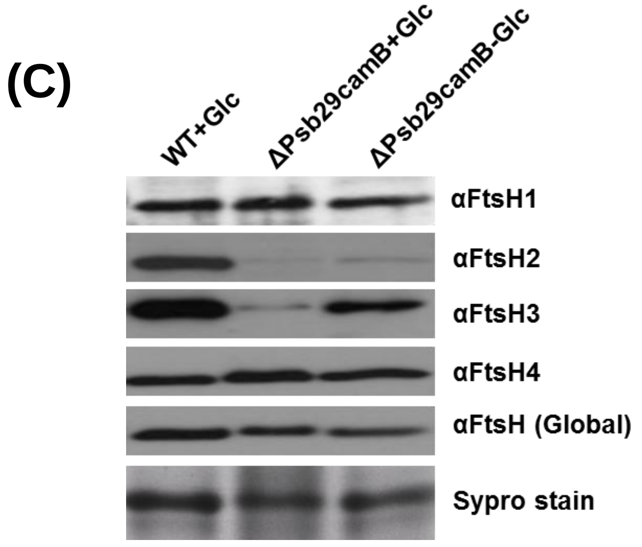

Fig. S1 Construction and characterisation of Psb29 null mutants
(A) Schematic representation of the vector construct used to disrupt *psb29*
(B) PCR analysis to confirm complete segregation of the mutants using primers to amplify the DNA fragments shown in (A)
(C) Immunoblot analysis of ΔPsb29camB grown in either the presence or absence of glucose (Glc) using antibodies specific for each of the 4 FtsH proteases plus a global FtsH antibody that recognises all FtsH subunits. Protein loading assessed by Sypro staining.

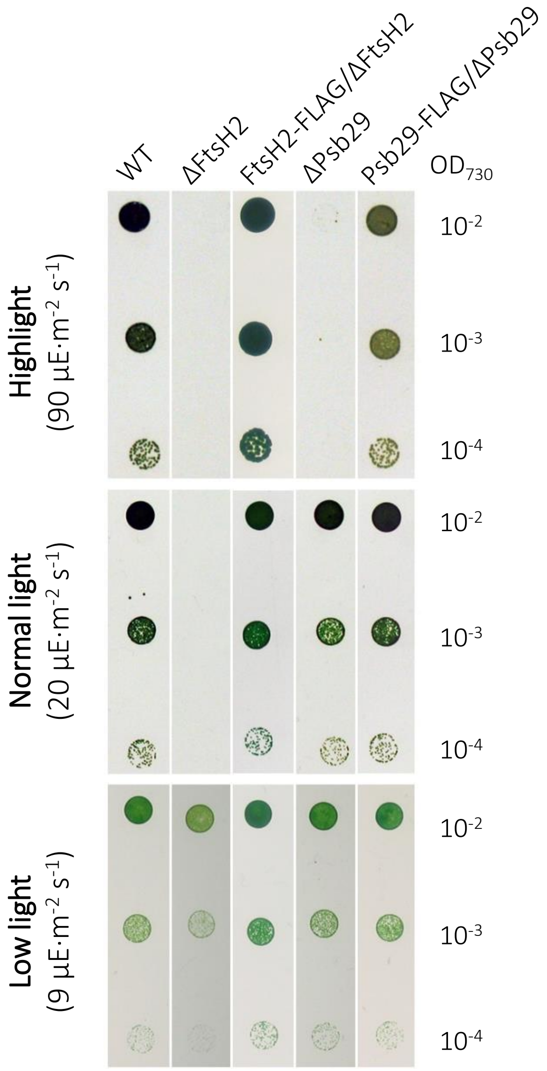

Fig. S2 Complementation of null mutants by FLAG-tagged derivatives

Growth assay of WT, ΔFtsH2, FtsH2-FLAG/ΔFtsH2, ΔPsb29 and Psb29-FLAG/ΔPsb29 cells diluted to OD_{730} of 10^{-2}, 10^{-3} and 10^{-4} on a BG11 agar plate, grown for 8 days at different light irradiences.

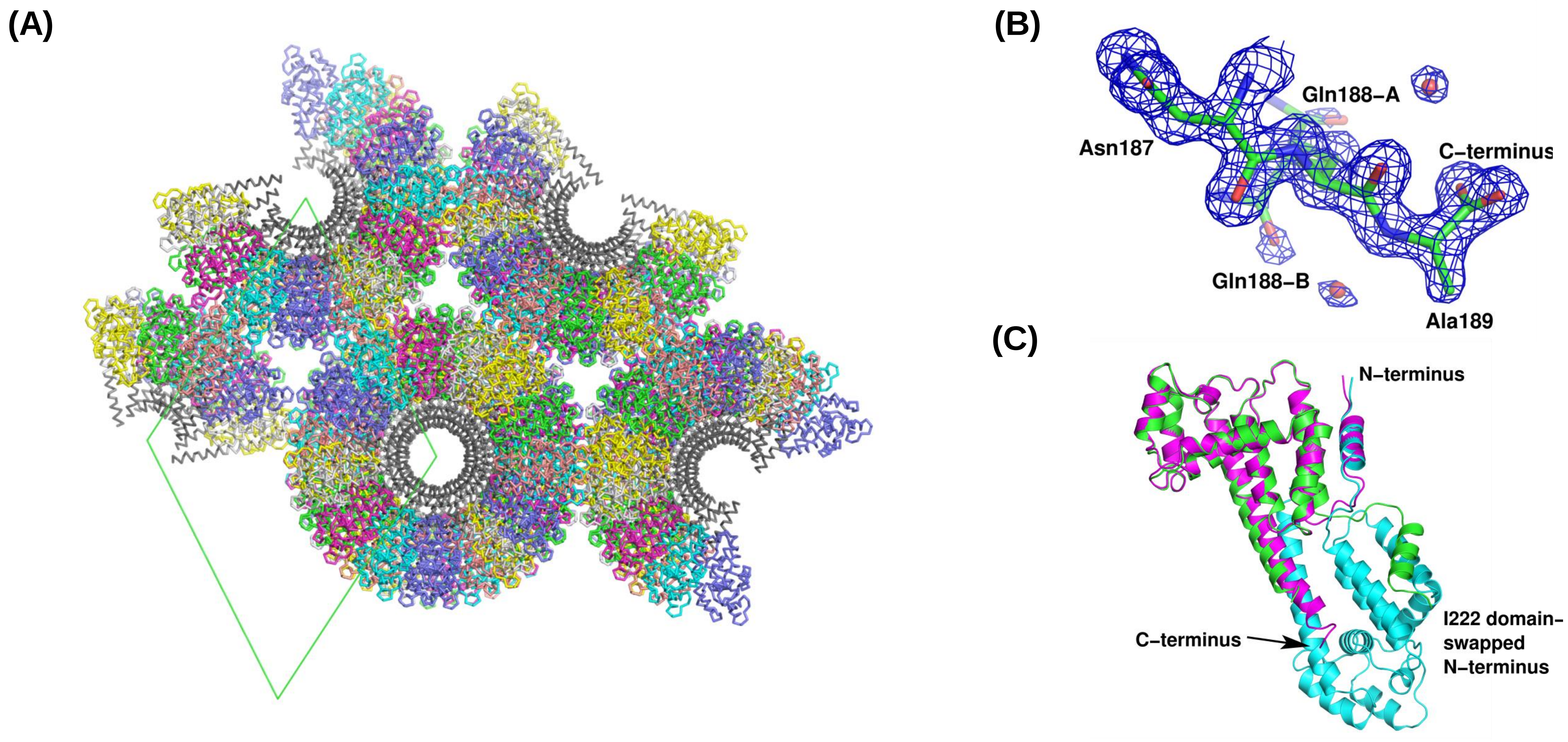

Fig. S3

(A) A view of part of the $P6_322$ crystal lattice, with the unit cell in green, looking down the c-axis. The Psb29 domains are shown in colour and the C-terminus from residue Arg190 in grey.

(B) The new C-terminus of the B-P21 crystal form at Ala189 with the 2mFo-DFc map contoured at 1σ. Gln188 is visible in two conformations (A and B) in the electron density.

(C) Cartoon showing the domain swap of the I222 crystal form in green compared to the $P6_322$ form in magenta. The domain swapped partner in the I222 crystal lattice (shown in cyan) provides the N-terminal helix

(A)

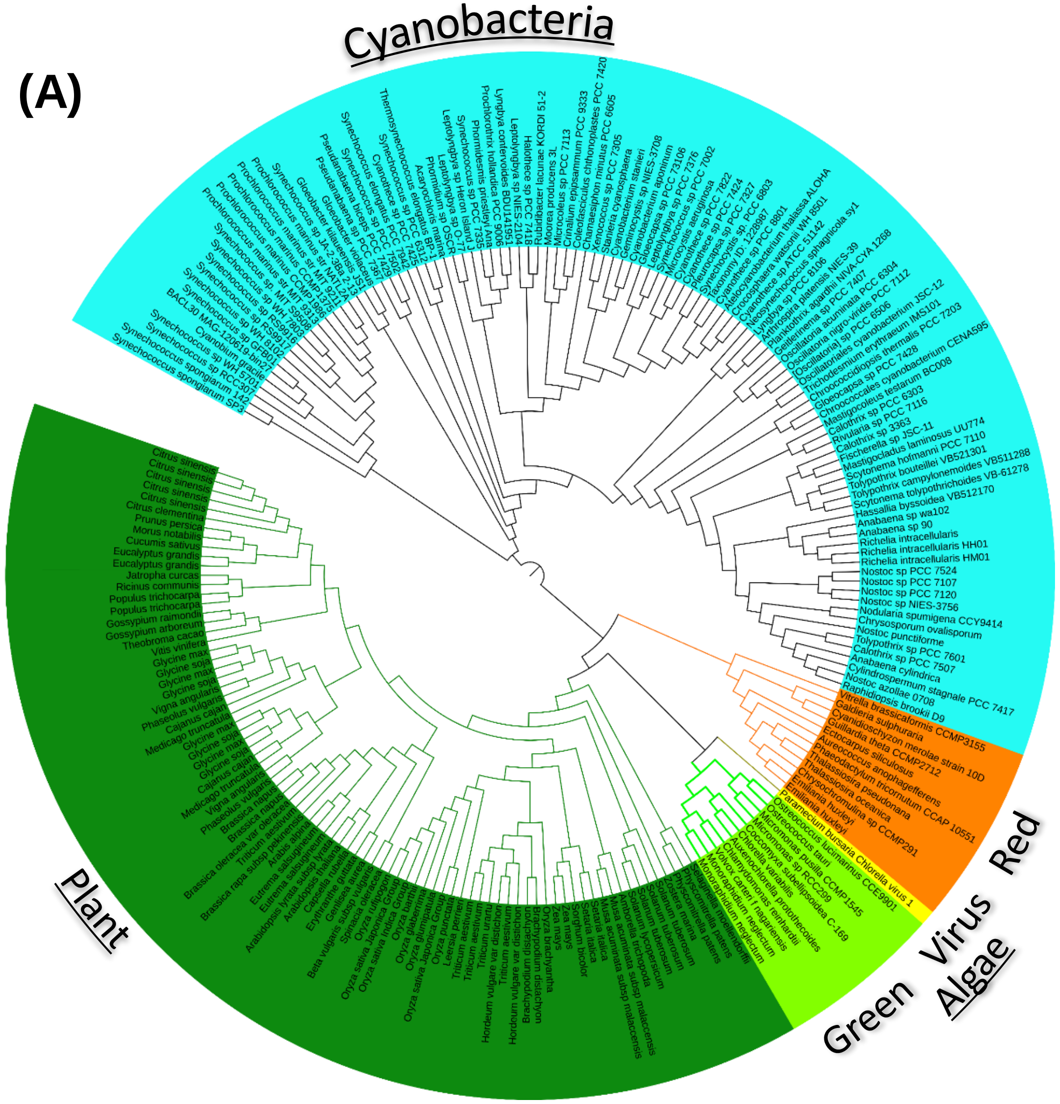

(B)

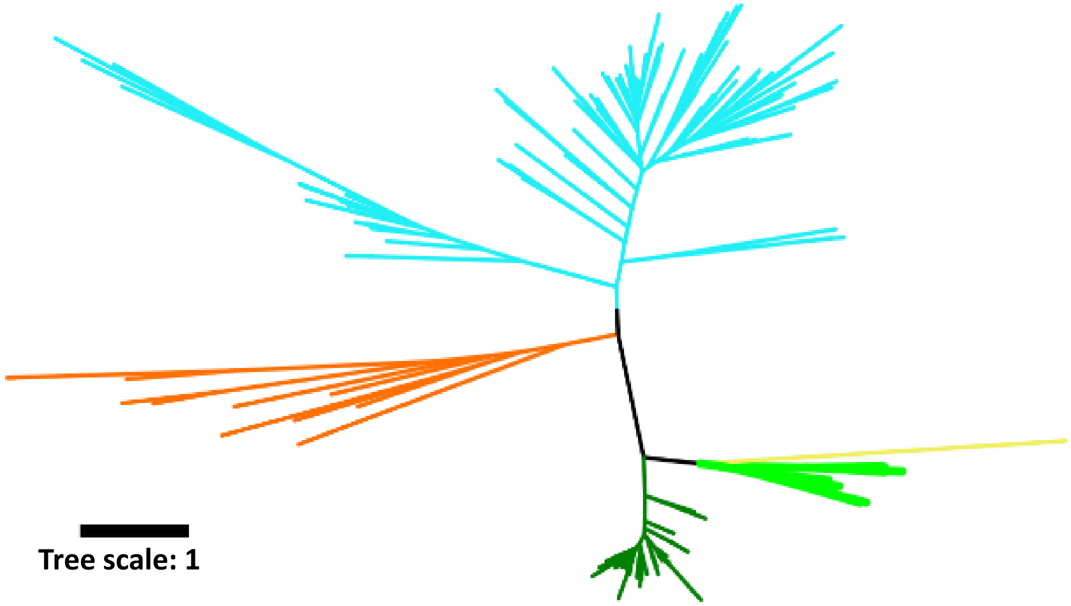

Fig. S4 A maximum-likelihood tree of Psb29/THF1

(A) Circular cladogram showing the phylogenetic relations of the 211 Psb29/THF1 sequences.

(B) Unrooted tree of Psb29/THF1. Colour scheme is: cyan – cyanobacteria; dark green – plant; light green – green algae; yellow – algal virus; orange – red algae.

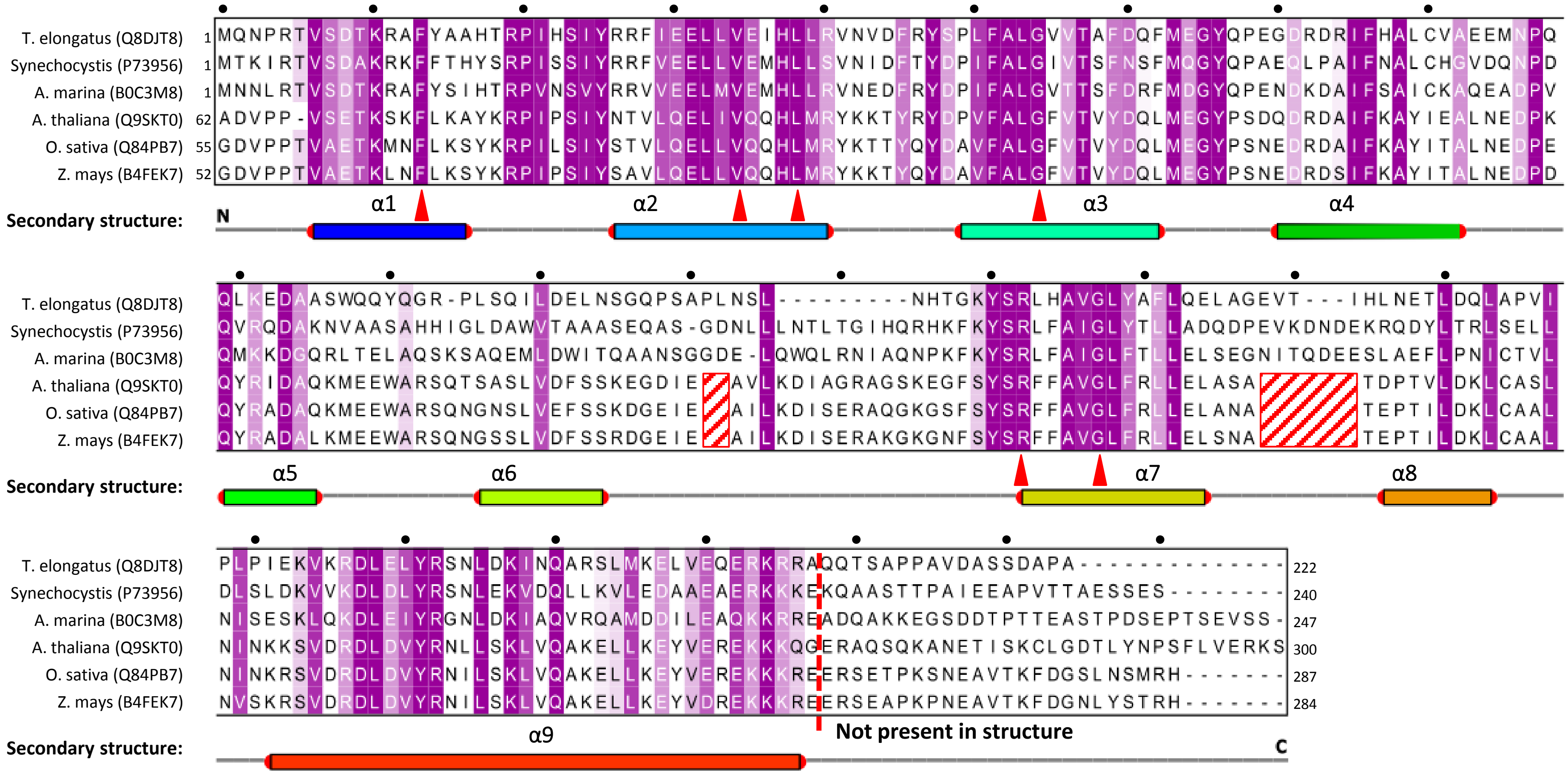

Fig. S5. Secondary structure of Psb29/THF1 and the comparison of cyanobacterial Psb29 and Plant THF1. Six representative Psb29/THF1 sequences (three cyanobacterial and three plant), as indicated by species name and the UniProt protein entry name, were selected and aligned by MAFFT then coloured by Jalview on the basis of sequence similarity (BLOSUM62 score). The background colour gradient from purple to white represents the similarity from high to low. Red striped rectangles show the position of two conserved insertion/deletions in the plant sequences. Underneath the alignment are the positions of the helices shown in Figure 3. Red triangles identify 100% conserved residues among 103 cyanobacterial and 84 plant Psb29/THF1 sequences surveyed in this study. Black dots indicate the sequence length.

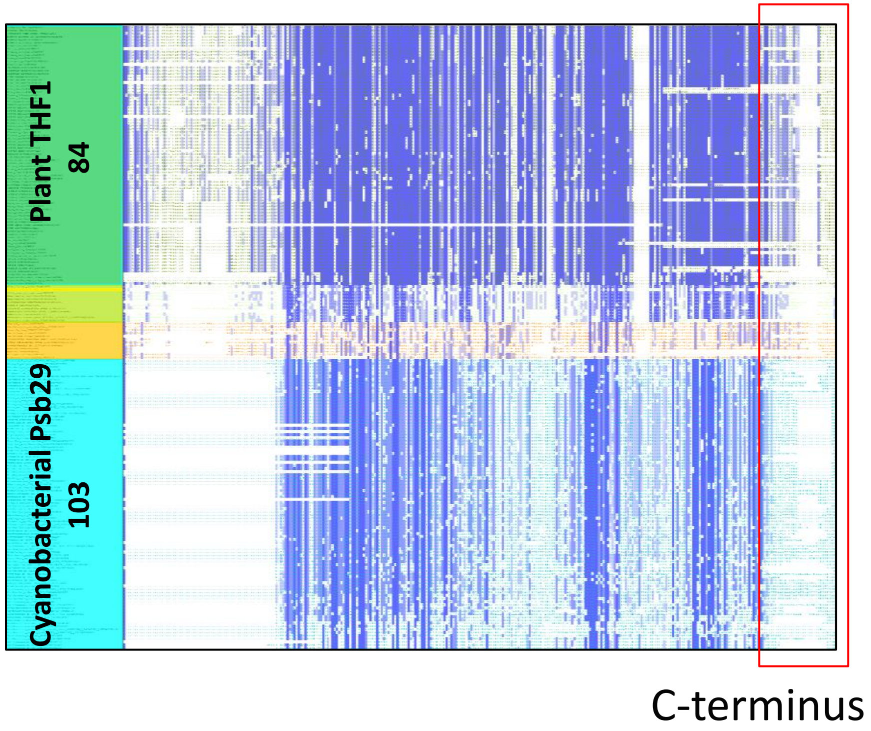

Fig. S6

Sequence comparison of all 211 Psb29/THF1 sequences. Different groups are coloured in different colour: Cyanobacteria, cyan; Red algae, orange; Green algae, light green; Virus, yellow; Dark green, plant. Column background colour represents percentage identity: Mid blue corresponds to >80% identity, light blue represents >60%, light grey represents >40% and white represents <40%.
